# Enhanced efficacy of AKT and FAK kinase combined inhibition in squamous cell lung carcinomas with stable reduction in PTEN

**DOI:** 10.18632/oncotarget.18087

**Published:** 2017-05-23

**Authors:** Andrea Cavazzoni, Silvia La Monica, Roberta Alfieri, Andrea Ravelli, Nele Van Der Steen, Rocco Sciarrillo, Denise Madeddu, Costanza Anna Maria Lagrasta, Federico Quaini, Mara Bonelli, Claudia Fumarola, Daniele Cretella, Graziana Digiacomo, Marcello Tiseo, Godefridus J. Peters, Andrea Ardizzoni, Pier Giorgio Petronini, Elisa Giovannetti

**Affiliations:** ^1^ Department of Medicine and Surgery, University of Parma, Parma, Italy; ^2^ Department of Medical Oncology, VU University Medical Center Amsterdam, Amsterdam, The Netherlands; ^3^ Medical Oncology Unit, University Hospital of Parma, Parma, Italy; ^4^ Division of Medical Oncology, Sant'Orsola-Malpighi University Hospital, Bologna, Italy; ^5^ Cancer Pharmacology Lab, AIRC/Start-Up Unit, University of Pisa, Pisa, Italy

**Keywords:** PTEN, FAK, PI3K, target therapy, squamous lung carcinoma

## Abstract

Squamous cell lung carcinoma (SCC) accounts for 30% of patients with NSCLC and to date, no molecular targeted agents are approved for this type of tumor. However, recent studies have revealed several oncogenic mutations in SCC patients, including an alteration of the PI3K/AKT pathway, i.e. PI3K point mutations and amplification, AKT mutations and loss or reduced PTEN expression. Prompted by our observation of a correlation between PTEN loss and FAK phosphorylation in a cohort of patients with stage IV SCC, we evaluated the relevance of PTEN loss in cancer progression as well as the efficacy of a new combined treatment with the pan PI3K inhibitor buparlisip and the FAK inhibitor defactinib. An increase in AKT and FAK phosphorylation, associated with increased proliferation and invasiveness, paralleled by the acquisition of mesenchymal markers, and overexpression of the oncomir miR-21 were observed in SKMES-1-derived cell clones with a stable reduction of PTEN. Notably, the combined treatment induced a synergistic inhibition of cell proliferation, and a significant reduction in cell migration and invasion only in cells with reduced PTEN. The molecular mechanisms underlying these findings were unraveled using a specific RTK array that showed a reduction in phosphorylation of key kinases such as JNK, GSK-3 α/β, and AMPK-α2, due to the concomitant decrease in AKT and FAK activation. In conclusion, the combination of buparlisib and defactinib was effective against cells with reduced PTEN and warrants further studies as a novel therapeutic strategy for stage IV SCC patients with loss of PTEN expression.

## INTRODUCTION

Within non-small-cell lung cancer (NSCLC), two major histology subtypes are adenocarcinoma (AD) and squamous cell carcinoma (SCC) accounting for 50–60% and 30% of cases, respectively. In the last decade encouraging new targeted treatments have improved the outcome of AD patients, whereas there are as yet no approved targeted therapies for the SCC histotype beyond standard chemotherapy, except the monoclonal anti-EGFR necitumumab, combined with cisplatin and gemcitabine. Unfortunately this regimen only led to a marginal survival benefit in EGFR immunohistochemical positive SCC [1]. Recently, three FDA-approved mAbs directed to the immune checkpoint PD-1/PDL-1 (nivolumab, pembrolizumab and atezolizumab) have been approved for NSCLC treatment [2–5]. Although these immunotherapies have generated great enthusiasm, clinical benefits are obtained in only 20% of patients, while the majority is refractory to these treatments. Therefore, in parallel with the identification of predictive biomarkers to tailor immunotherapeutic agents, further research on new oncogene-directed targeted drugs is needed.

The Cancer Genome Atlas Project revealed several genes commonly altered in SCC, including phosphatidylinositol-3-OH kinase (PI3K) [6]. The PI3K/AKT/mTOR pathway is negatively regulated by the tumor suppressor gene *Phosphatase and tensin homologue* (*PTEN*), which maps on chromosome 10q23 and encodes for a lipid phosphatase that converts the PI3K product PtdIns (3, 4, 5)P3 to PtdIns (4, 5)P2. PtdIns (3,4,5)P3 recruits AKT and PDK1 kinases at the plasma membrane enabling AKT phosphorylation of the Thr308 residue by PDK1 and of Ser473 by mTORC2 complex. Active AKT drives cell survival, cell proliferation, angiogenesis and cellular metabolism by phosphorylating downstream signaling proteins, including glycogen synthase kinase 3 (GSK3), forkhead box O (FOXO), B cell lymphoma 2 (BCL2), antagonist of cell death (BAD), the E3 ubiquitin-protein ligase MDM2 and p27 [7].

There is increasing evidence for a critical role of PTEN protein in NSCLC progression, and abrogation of PTEN function may occur through multiple mechanisms. *PTEN* is a commonly altered tumor suppressor gene in human lung cancers [8], and immunohistochemical analysis demostrated that PTEN levels are reduced in 70 and 77% of patients with SCC and AD hystologies, respectively [9]. Loss of PTEN expression may be a consequence of mutation, deletion, decreased protein synthesis, elevated protein degradation or turnover, or other post-translational modifications. Another possible mechanism is the epigenetic inactivation of the gene via hypermethylation of the promoter region [10] or by microRNA (miRNA) regulation.

It has been demonstrated that miR-21, a well-known oncomir, is overexpressed in a number of malignancies including lung cancer. Importantly, this miRNA regulates the expression of PTEN by directly targeting its 3′ untranslated region (3′UTR) and therefore reducing *PTEN* mRNA translation [8].

The central role of PTEN inactivation in tumor development and progression is related not only to AKT activation, but also to increased phosphorylation of another PTEN target, the focal adhesion kinase FAK. PTEN can indeed interact with and dephosphorylate FAK, leading to the inhibition of integrin-mediated cell spreading, migration and invasion [11, 12]. FAK dephosphorylation by PTEN has been documented in human T-cell acute lymphoblastic leukemia, glioblastoma, colorectal, uterine and gastric cancers [11–15]. Moreover, an interaction of these proteins has been documented by Tzenaki and collaborators [13] in breast cancer cells; in particular, it has been reported that PTEN phosphorylation at Tyr336 by FAK is a critical event for its phosphatase activity, demonstrating the relevance of a critical loop between PTEN and FAK proteins. Increased expression of FAK kinase was documented in lung cancer [14], especially in advanced stage, suggesting its potential involvement in disease progression.

In this study, prompted by our observation that most patients with metastatic SCC harbored PTEN downregulation, associated with increased FAK phosphorylation, we propose a new combined treatment with the pan-PI3K inhibitor buparlisib and the FAK inhibitor defactinib in SCC cells with low PTEN levels. This combination was tested in stable cell clones from SKMES-1 cells, that we generated using synthetic miRNA directed against PTEN mRNA. These clones were characterized in term of cell viability both in two-dimensional (2D) monolayer cultures and in three-dimensional (3D) systems, as well as for cell migration and invasion. We demonstrated a synergistic effect of the combination of buparlisib and defactinib in cells with low PTEN levels, whereas was absent in cells carrying activating mutations of PI3K enzyme [15]. These results demostrated that PTEN abrogation is a potential predictive factor for the rational use of a combined treatment of PI3K inhibitors with FAK inhibitors in SCC.

## RESULTS

### Correlation between PTEN downregulation and FAK activation in SCC patients

A total of 51 SCC patients with resected (n 25) or metastatic disease (n 26) were analyzed. The clinicopathological characteristics of the patients are presented in Supplementary Table 1. The median age was 71 years (range 47–85). The majority of patients were male (88%) and current or ex-smokers (82%). Most patients (51%) had metastatic disease (stage IV) at diagnosis; from the 49% remaining patients, 29% were resected and remained disease-free, whereas the other 20% patients, despite surgery, suffered from relapse. In particular, pathological analyses revealed stage I in 7.8 %, stage II in 15.7% and stage III in 5.9% of the tissues from the resected and disease-free groups; in the relapsed group, 7.8% of the patients had stage I, 5.9 stage II and 5.9 % stage III.

**Figure 1:**
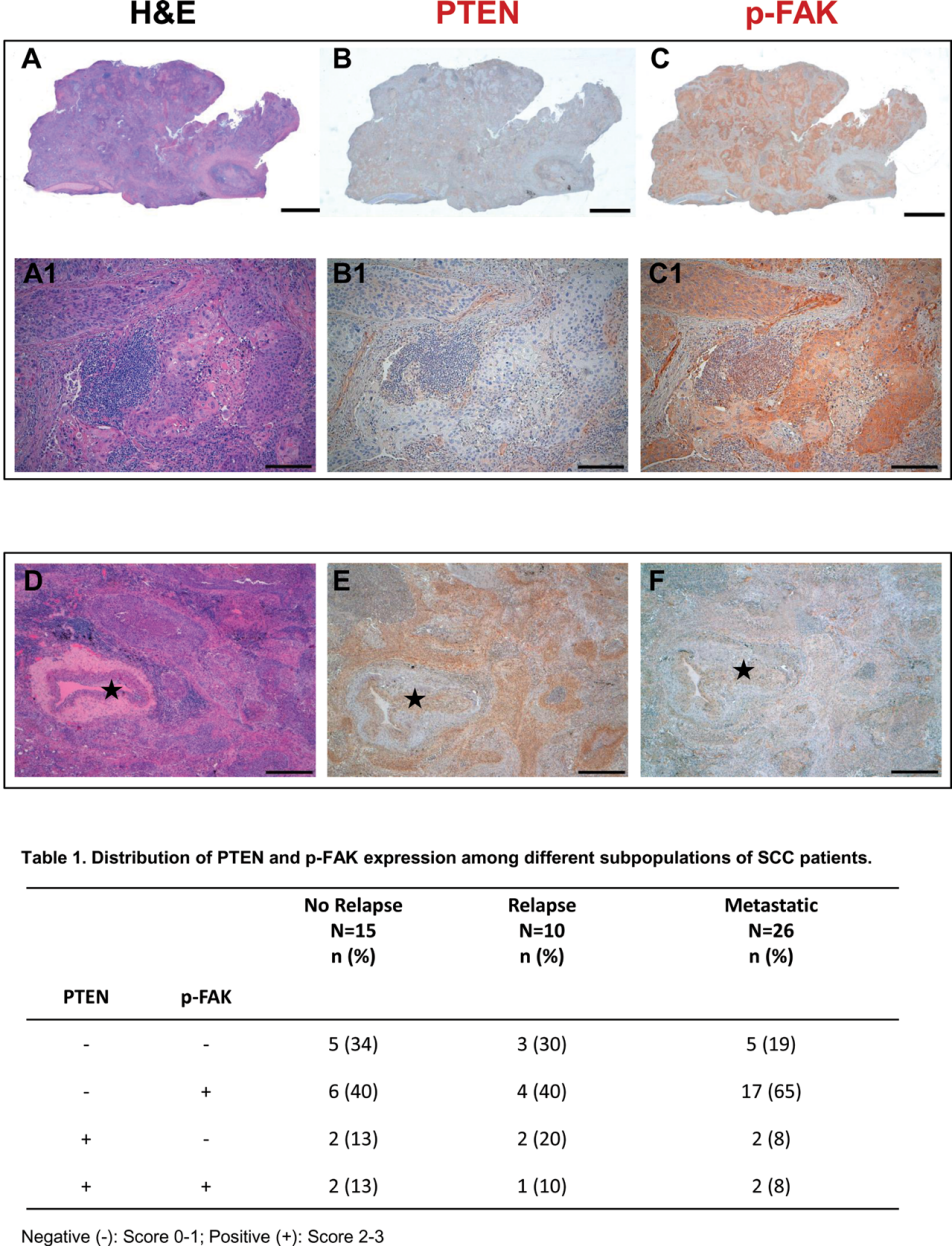
PTEN and phospho-FAK are negatively correlated in metastatic SCC cancer samples Serial histologic sections of a representative human SCC biopsy stained by Hematoxylin-Eosin (**A**) and immunostained with specific antibodies to document the absence of PTEN expression (**B**) and the presence of p-FAK (**C**, brownish) in neoplastic tissue. Higher magnification of the same samples are shown on corresponding panels in A1–C1. (**D**–**F**) Serial sections of a SCC sample to illustrate in the same microscopic field stained by Hematoxylin-Eosin (D), PTEN positive (E, brownish) and p-FAK negative (F) cancer cells surrounding an arteriole (⋆). Scale bars: (A–C)2 mm; (A1–C1) 200 μm; (D–F) 500 μm.

As shown in Figure 1, high PTEN levels (score 2–3) were detectable in 26% of disease free, 30% of relapsed groups, and only 16% of metastatic diseases, whereas 84% of the metastatic patients presented null or low (score 1) PTEN expression. Furthermore, FAK phosphorylation at Tyr925 was detected in 53% of patients (resected and disease-free) and 50% of patients from resected and relapsed group, while the metastatic group presented high levels of FAK activation. Altogether, 65% of patients with metastatic disease presented with PTEN downregulation, associated with increased FAK phosphorylation.

### Effect of PTEN silencing on cell proliferation and spheroid formation

**Figure 2:**
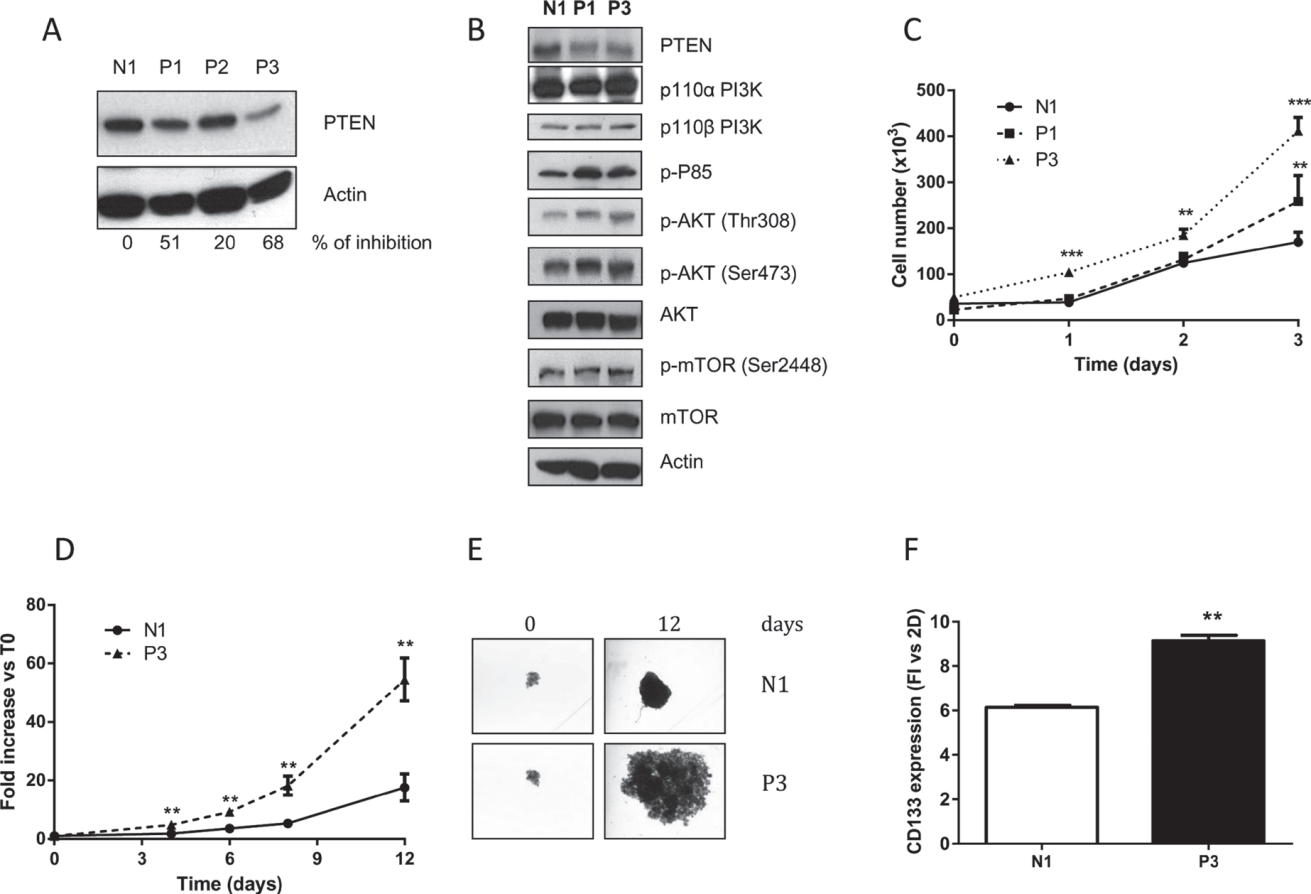
Reduction of PTEN increases AKT phosphorylation and growth rate in 2D and 3D models (**A**) Clones generated from SKMES-1 cells after transfection with a pool of four different miRNAs directed to PTEN mRNA were tested for PTEN protein level. The cells were lysed and levels of PTEN and β-actin were determined by immunoblotting with the specific antibodies for the targets reported in each row. The immunoreactive spots were quantified by densitometric analysis, and the calculated values for the ratio of PTEN/Actin were expressed as % of inhibion of PTEN expression. (**B**) Western blotting showing levels of PTEN, PI3K subunits p110 α and β, p85, AKT and mTOR in total lysates from N1 and clones P1 and P3. (**C**) Growth rate curves of N1 and P1-P3 clones at the indicated time intervals. (**D**) At the indicated times, the volume of spheroids from N1 and P3 was measured and the Fold Increase (FI) index was calculated as the ratio between the spheroid volume after 4–6–8 and 12 days and the volume at T0. (**E**) Representative images of spheroids from N1 and P3 clones at T0 and after 12 days of culture. (**F**) Expression level of CD133 protein in spheroids from N1 and P3 clones was evaluated by flow cytometry and the quantification is reported as fold increase versus 2D for N1 and P3 cells. The results in A, B, E are from representative experiments. Each experiment, repeated three times, yielded similar results. Experiments in C and D are the mean value (±SD) of three independent measurements. **p < 0.01; ***p < 0.001.

SKMES-1 cells were transfected with a pool of four plasmids each carrying a different synthetic miRNA directed against PTEN mRNA; after antibiotic selection and screening, we obtained three clones with stable reduction of PTEN levels (P1, P2 and P3). As shown in Figure 2A, significant PTEN reduction was documented in P1 and P3 clones, with a percentage of PTEN inhibition of 51% and 68% versus N1 clone (transfected with empty vector), respectively. Therefore, we used these two models for the following pharmacological and mechanistic studies. Figure 2B shows the levels of different proteins involved in the PI3K/AKT/mTOR pathway, and highlights an overall increase in the levels of phosphorylation of AKT in both the serine and threonine residues, as well as of p-p85, the regulatory subunit of PI3K α and β. The effect of PTEN silencing on cell proliferation was then evaluated, and we detected a clear increase in cell number at regular time intervals for the transfected clones with respect to the parental cells (Figure 2C).

Since earlier studies showed that multicellular spheroid models might be especially useful to evaluate the molecular consequences of PTEN deletion [16], we also evaluated the effect of PTEN abrogation on spheroid growth, using the clone with the highest inhibition of PTEN (P3): the growth of spheroids was significantly enhanced in P3 cells, compared to N1 cells (Figure 2D–2E). In addition, we collected and used the spheroids for flow cytometry evaluation of the cancer stem cells (CSC) marker CD133, which has also been correlated with metastatic spread [17]. As shown in Figure 2F, the detected levels of CD133 were significantly increased in N1 and P3 cell clones grown in 3D compared to 2D models; moreover CD133 was expressed at higher levels in P3 spheroid cell culture compared to N1 spheroids, supporting the presence of a higher percentage of cells with a CSC-like phenotype.

### Effect of PTEN down-regulation on cell migration and invasion

**Figure 3:**
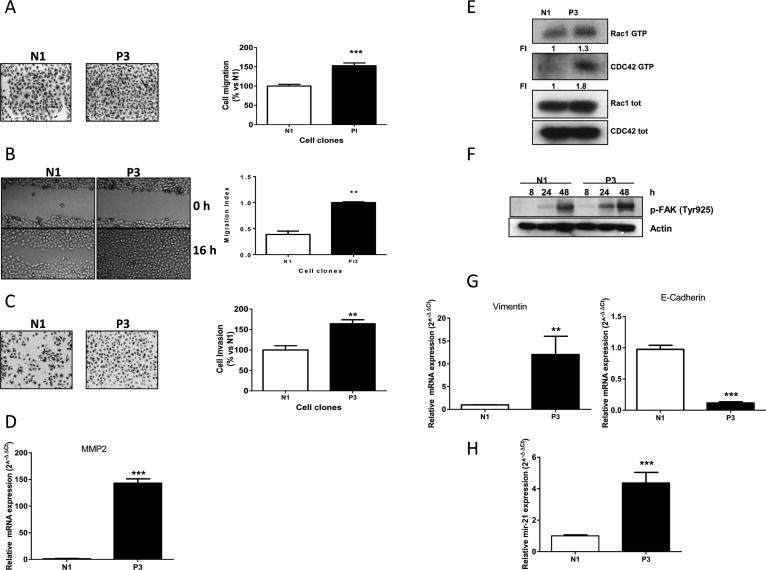
Increased migration, invasion properties and EMT induction in cells with low PTEN levels (**A**) Representative images and analysis of migrated cells in N1 and P3 clones after 16 h. Columns, means of 10 fields counted; bars, SD. (**B**) Representative images and analysis of wound-healing assay in N1 and P3 cells after 0 and 16 h. (**C**) Representative images and analysis of invaded cells in N1 and P3 clones after 16 h. Columns, means of 10 fields counted; bars, SD. (**D**) MMP2 expression in N1 and P3 clones evaluated by Real Time PCR. (**E**) Determination of RAC1 and CDC42 activities in N1 and P3 clones by pull-down assay. (**F**) Western blotting showing FAK activation in both clones at the indicated time points. Cells were plated in culture dishes and after cell adhesion (24 h) culture medium was changed with fresh medium without drugs. At indicated time intervals cell lysis was performed. (**G**) Evaluation of the EMT markers Vimentin and E-cadherin by Real Time PCR in N1 and P3 clones. (**H**) Determination of miR-21 expression levels in both clones by Real Time PCR. The results in E and F are from representative experiments. Each experiment, repeated three times, yielded similar results. Experiments in A, B, C, D, G and H are the mean value (±SD) of three independent measurements. **p < 0.01; ***p < 0.001.

Since previous studies suggested a key role of PTEN in the invasive behavior of different cancer types [16, 18] we evaluated the effect of PTEN abrogation on cell migration and invasion in our SCC models. These studies revealed that reduced PTEN expression was clearly associated with an enhanced migration potential (Figure 3A). Furthermore, P3 cells spread into the wound area more efficiently than did the N1 clone, as shown and quantified in Figure 3B. We then investigated the effect of PTEN on the invasiveness of the P3 and N1 cell clones. Figure 3C shows that P3 cells had higher invasivity than did the N1 ones. Interestingly, this increased invasive behaviour was associated with abnormally higher levels of matrix metalloproteinase-2 (MMP2) expression when PTEN was down-regulated (Figure 3D).

Since cell motility and cytoskeletal rearrangement are two processes known to be predominantly regulated by the PI3K/AKT/mTOR pathway, we performed a pull down assay measuring the expression levels of RAC1 and CDC42 both as active GTP-binding form and in total. As can be seen in Figure 3E, the presence of the active form of both proteins was clearly increased in the P3 cells compared to levels in N1 clone. Similarly, we also investigated the level of phosphorylation of FAK as PTEN normally acts as a phosphatase on FAK [11, 12]. In particular, we analyzed the levels of Tyr925, a phosphorylation site of FAK, at different time intervals (8, 24, 48 hours), finding a relevant increase of phosphorylation levels, as a consequence of increase of cell adhesion and cell-to cell interaction [19] (Figure 3F). The same result was obtained for the other critical residue of FAK phosphorylation at Tyr327 (data not shown).

As a measure of the metastatic potential of P3 clone, we analyzed the mRNA expression levels of Vimentin and E-cadherin, two markers typically associated with epithelial-to-mesenchimal transition (EMT) features. As expected, we found that higher Vimentin expression levels and reduced expression of E-cadherin were associated with the P3 clone as compared to the control N1, revealing a link between PTEN down-regulation and EMT features (Figure 3G). Finally, we also found an association between PTEN down-regulation and increased levels of miR-21 (Figure 3H), an oncomir that has been proposed to impact cancer progression, also through PTEN regulation [8, 20].

### Effect of buparlisib and defactinib on N1 and P3 cell clone proliferation

**Figure 4:**
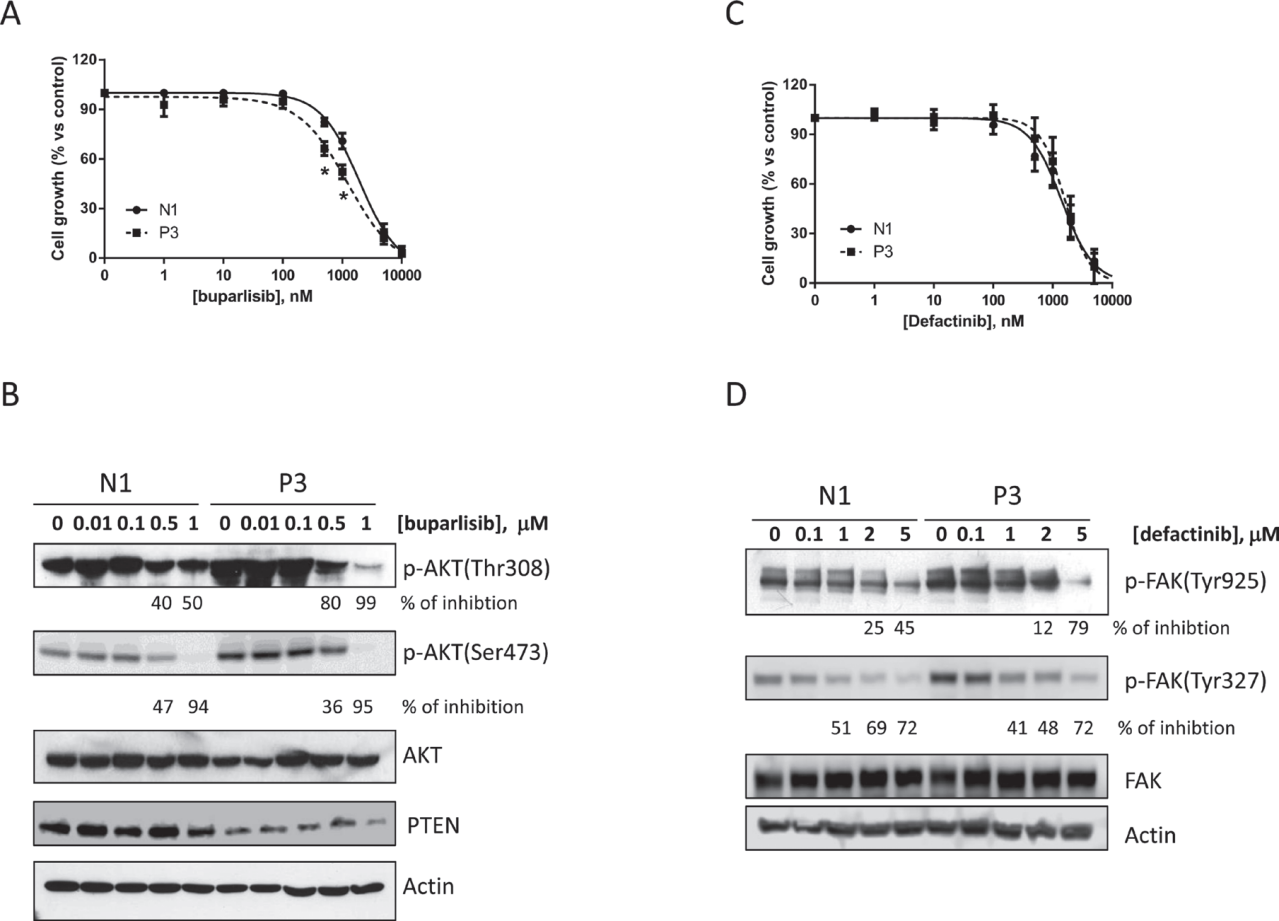
Buparlisib and defactinib have similar effects in inhibiting cell growth in N1 and P3 cells (**A**) N1 and P3 cells were treated with increasing concentrations of the pan PI3K inhibitor buparlisib and cell growth was determined by SRB assay. (**B**) Western blot analysis of AKT phoshporylation in N1 and P3 cells treated for 8 h with buparlisib at the indicated dose range. (**C**) N1 and P3 cells were treated with increased concentrations of the FAK inhibitor defactinib and cell growth was determined by SRB assay. (**D**) Western blot analysis of FAK phosphorylation in N1 and P3 cells treated for 24 h with defactinib at the indicated dose range. The results in B and D are from representative experiments. Each experiment, repeated three times, yielded similar results. Experiments in A and C are the mean value (± SD) of three independent measurements. *p < 0.05.

After the establishment of cell clones with inhibition of PTEN that showed differential biological features, we investigated the anti-proliferative effect of buparlisib, a potent class I pan-PI3K abrogating agent, in the P3 compared to N1 clones (Figure 4A). Although we observed a certain grade of inhibition in both cell clones, the anti-proliferative effect of this compound was weakly evident in the P3 clone at the critical concentrations of 0.5 and 1 μM, whereas at higher concentrations the similar activity of buparlisib in P3 and N1 were presumably related to PI3K-independent effects of the drug [21]. Accordingly, the level of phosphorylation of AKT was decreased after treatment with buparlisib in the P3 cell clone (Figure 4B) at lower concentrations than in N1 cells (i.e., 0.1 μM) only for the Thr308 residue and not for the Ser473 residue. Moreover PTEN expression was not altered by buparlisib treatment.

Similarly, the increased phosphorylation of FAK prompted us to evaluate the effect of defactinib, a novel FAK inhibitor, on the proliferation of N1 and P3 cell clones. As shown in Figure 4C, 4D, the proliferation assay and the phosphorylation analysis of FAK did not display any significant difference between the two cell clones after defactinib treatment (Figure 4D).

### Effect of the combination of buparlisib with defactinib on cell growth and migration

**Figure 5:**
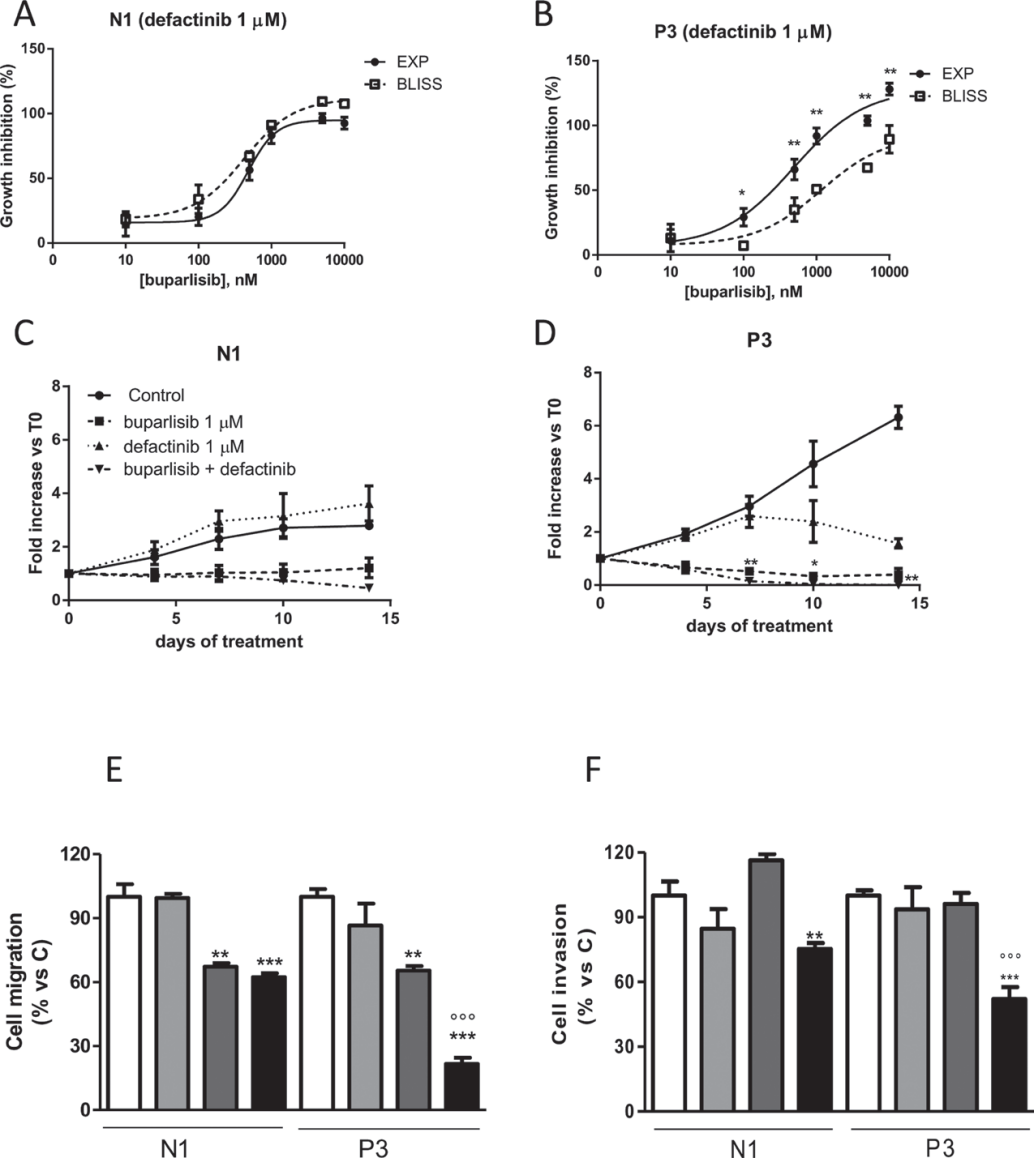
The combination of buparlisib and defactinib showed synergistic effect in term of cell viability, cell migration and invasion only in cells with reduced PTEN levels (**A**, **B**) Curves of the growth-inhibitory effects of the combined treatment of buparlisib with defactinib (1 μM) versus theoretical Bliss additivity curve for N1 and P3. Cells were treated with the drugs for 3 days and then cell growth was assessed using SRB assay staining as described in Materials and Methods Section. Data are expressed as percent inhibition of cell proliferation versus control cells. The experiments, repeated three times, yielded similar results. *p < 0.05; **p < 0.01.(**C**, **D**) The volume of spheroids from N1 and P3 clones untreated or exposed to 1 μM buparlisib or 1 μM of defactinib or their combination was measured and the Fold Increase (FI) index was calculated as the ratio between the spheroid volume at indicated time intervals and the volume at T0. *p < 0.05; **p < 0.01 vs buparlisib alone. (**E**) Representative images of spheroids from N1 and P3 clones at T0 and after 14 days of culture after treatment with the indicated doses of compounds. (**F**) Evaluation of migration in N1 and P3 clones after 16 h of treatment with buparlisib and defactinib at indicated concentrations. Columns, means of 10 fields counted; bars, SD. (G) Analysis of invaded cells in N1 and P3 clones after 16 h of treatment with buparlisib and defactinib. Columns, means of 10 fields counted; bars, SD. Each experiment, repeated three times, yielded similar results. Experiments in A and C are the mean value (±SD) of three independent measurements. *p < 0.05, ***p < 0.001 vs Control; ^###^*p* < 0.001 vs buparlisib; °°°*p* < 0.001 vs defactinib; ^$$^*p* < 0.01,^$$$^*p* < 0.001 vs N1 combination.

Since buparlisib or defactinib alone appeared to be weakly effective in both clones, we investigated the effect of a combined treatment. Specifically, we performed a dose-response curve of buparlisib in the presence of constant 1 μM concentration of defactinib. To evaluate the nature of the interactions, we used the Bliss interaction model. As shown in Figure 5A, the combination of the two compounds induced a weak additive effect on the inhibition of N1 cells growth, but was synergistic in P1 and P3 clones (Supplementary Figure 1 and Figure 5B). To confirm the efficacy of this drug combination, we also calculated the combination index CI [22]. As shown in Supplementary Figure 2, the concomitant drug administration resulted in a synergistic effect in P3 cells (CI<1), whereas an additive-antagonistic interaction was observed in N1 cells, with CI values above 2. Similar results were found for the other clone P1 (data not shown).

To further investigate the effect of this combinatory regimen, we analyzed the growth of 3D models in the presence of individual drugs or their combination. As shown in Figure 5C–5D, P3 cell spheroids were more sensitive to both drugs. Although N1 and P3 cell clones were sensitive to the combination, this treatment was more effective in the presence of PTEN down-regulation. Indeed, compared to P3 cells exposed to individual agents, cells treated with buparlisib/defactinib combination showed remarkably reduced formation and increased disintegration of multicellular spheroids, which were significantly smaller. Similar results were found for the other clone P1 (Supplementary Figure 3A–3B). In addition, when cells from spheroids were re-plated in adherent plates at the end of treatment, all cells were viable except those from P3 spheroids treated with the combination (data not shown). Considering that AKT and FAK pathways are involved not only in cell proliferation but also in cell migration and invasion, we evaluated the ability of individual or combined targeted inhibition to affect migration and invasiveness. As shown in Figure 5E, 5F, the combination demonstrated a potent inhibitory effect both on cell migration and invasion, with significantly stronger effects in the P3 cell clone compared to N1 control cells. Similar results were obtained for the other clone P1 (Supplementary Figure 4).

In *vivo* experiments showed that the effect of the combination was statistically significant different compared to control and defactinib treatment (Supplementary Figure 5A); however, we observed only a trend toward statistical significance for the combination treatment compared to buparlisib. This might be explained by the fact that in the combination we used relatively low doses, which were selected in order to avoid toxic effects. Indeed, as shown in the Supplementary Figure 5B, both single regimens and combination treatment do not cause reduction of animal body weight compared to untreated animals, demonstrating that tumor shrinkage induced by combination treatment was not accompanied by relevant toxicity.

### Effect of buparlisib, defactinib and their combination on a selected panel of phospho-kinases

**Figure 6:**
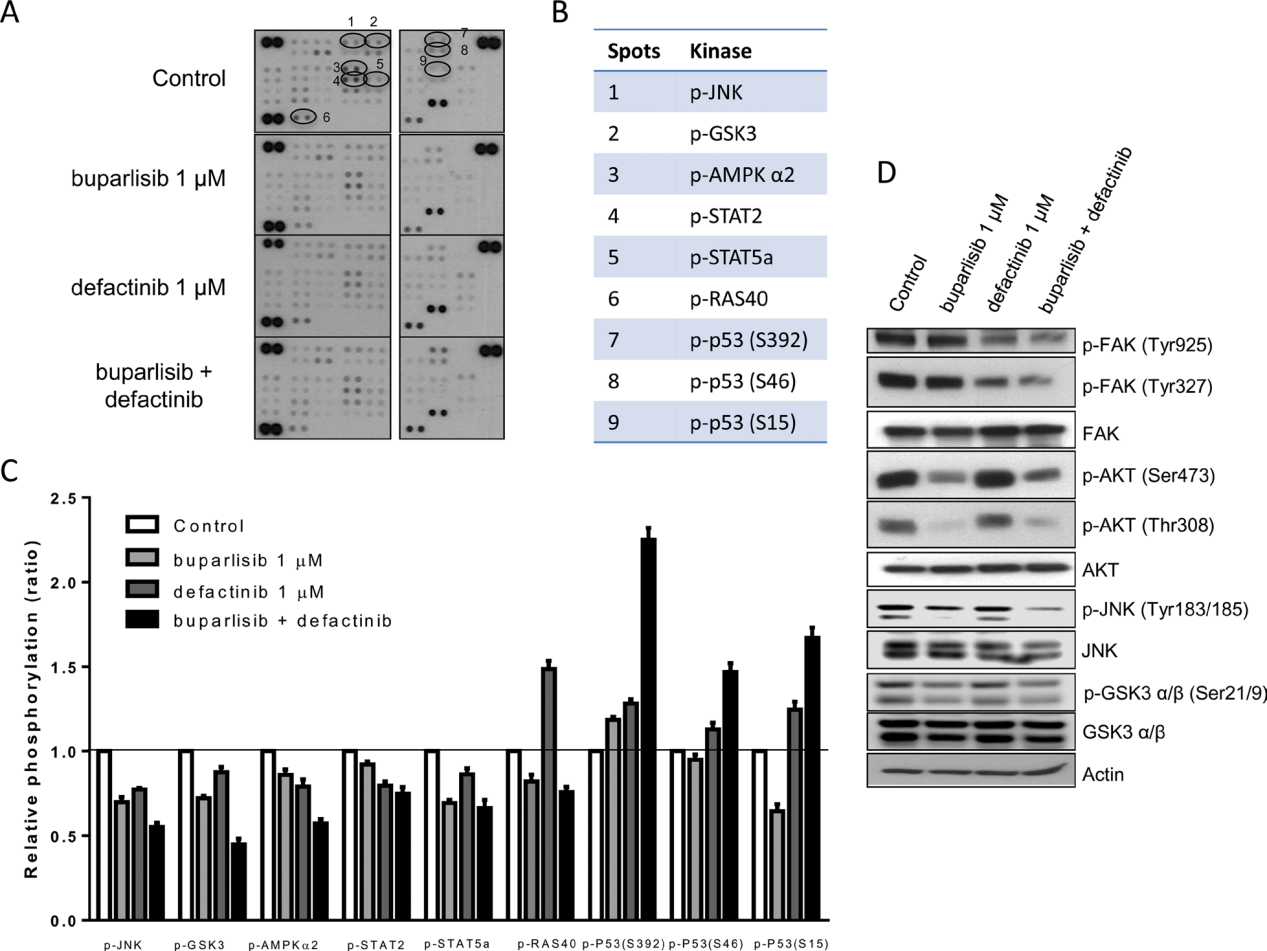
Combination of buparlisib and defactinib reduced phosphorylation of critical intracellular kinases (**A**) P3 cells were treated with the indicated doses of drugs for 24 h and lysates were incubated with human phospho-kinase array membranes and bound phospho-proteins were detected according to kit instructions. Each membrane contains specific kinase and positive control antibodies spotted in duplicate. (**B**) Six proteins (JNK, GSK3, AMPK-α2, STAT2, STAT5a, RAS40) exhibited a significant decrease in their phosphorylation status and p53 protein exhibited increased phosphorylation at three different serine residues following combination treatment vs single doses. (**C**) Columns, means of relative levels of protein phosphorylation of duplicate spots from a single experiment. (**D**) Western blot analysis of lysates from P3 cells treated with buparlisib, defactinib or combination for 24 h at indicated concentrations. Experiments in A are the mean values (±SD) of two independent measurements. The results in D are from a representative experiment.

To better investigate the signaling pathways specifically affected by the concomitant use of buparlisib and defactinib, 43 specific Ser/Thr or Tyr phosphorylation sites of 35 different proteins were analyzed by a human phospho-antibody array (Figures 6A, 6B). Nine phosphorylation sites exhibited a significant alteration after the co-administration of buparlisib and defactinib of P3 cells, compared to single regimen (Figure 6C). In particular, the combination induced a significant inhibition of JNK, GSK3 and AMPKα2 phoshorylation over single-drug administration. A certain grade of inhibition of the phosphorylation rate of STAT2, STAT5a and PRAS40 was also recorded. Conversely, the phosphorylation of p53 on Ser392, Ser46 and Ser15 domains were significantly increased when buparlisib and defactinib were administered in combination, compared to monotherapy.

As shown by the Western blot analysis (Figure 6D), the combination of buparlisib and defactinib was associated with a strong reduction in the levels of FAK phosphorylation of both Tyr925 and Tyr327 residues, and of JNK phosporylation (Tyr183–185) compared to single drugs. Instead the reduction in AKT phosphorylation of both Ser473 and Thr308 and of GSK3α/β was comparable to the effect observed after exposure to buparlisib.

### Effect of buparlisib and defactinib treatment in cell clones with aberrant expression of PI3K

**Figure 7:**
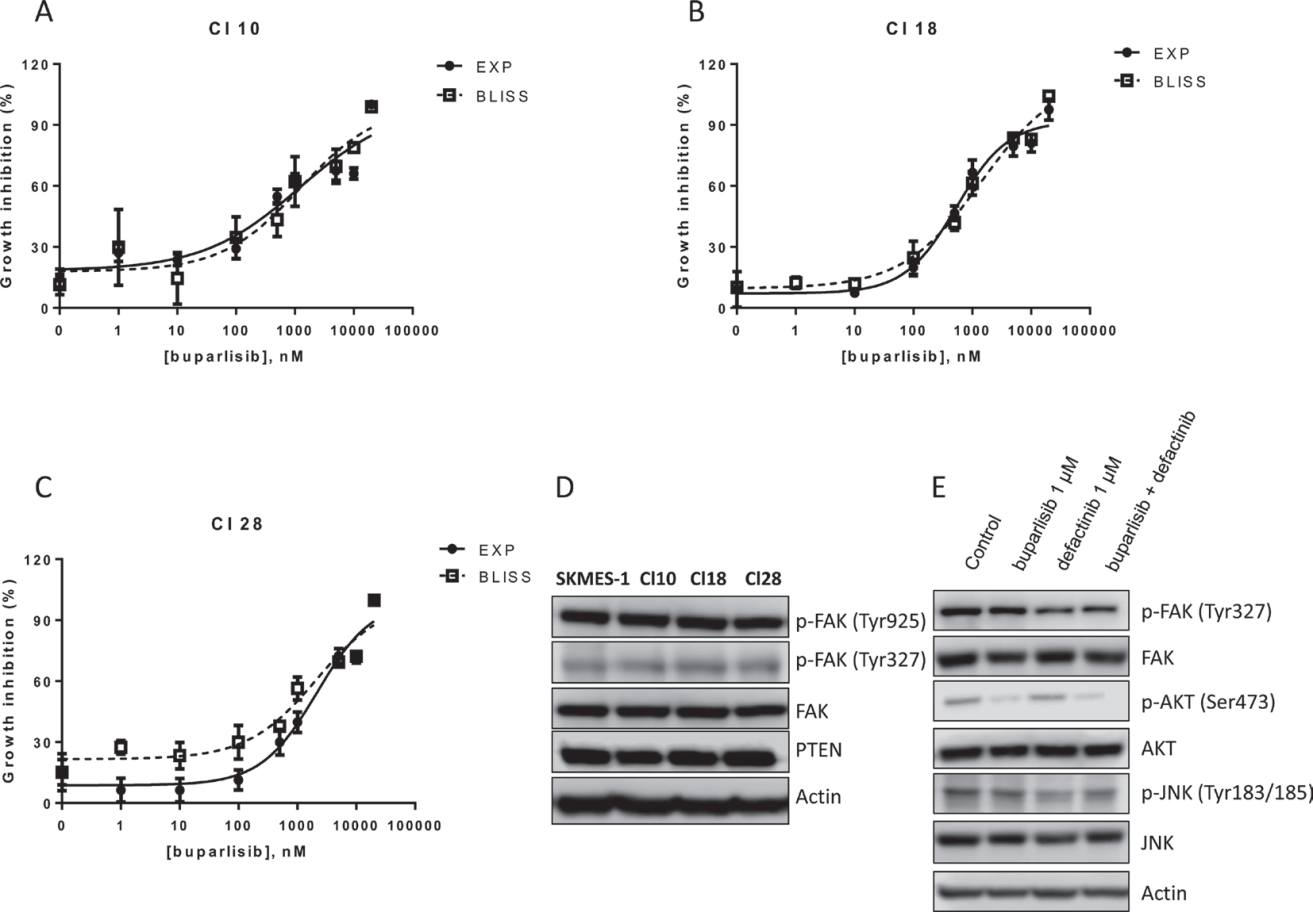
Combination of buparlisib and defactinib is additive in SKMES-1 derived clones with PI3K activating mutations (**A**–**C**) Curves of growth-inhibitory effects of buparlisib and defactinib combined treatment versus theoretical Bliss additivity curve were reported for Cl 10, Cl 18 and Cl 28. In the combined treatment, defactinib was 1 μM. Cells were treated with the drugs for 3 days and then cell growth was assessed using SRB assay staining as described in Materials and Methods Section. Data are expressed as percent inhibition of cell proliferation versus control cells. The experiments, repeated three times, yielded similar results. (**D**) Western blot analysis of lysates from CI 10, Cl 18 and Cl 28 clones. Cells were seeded and after 24 h the medium was changed and maintened for 1 day. At the end, cells were lysed and the indicated protein levels were detected by Western blot analysis. (**E**) Western blot analysis of lysates from Cl 18 cells treated with buparlisib, defactinib or combination for 24 h at indicated concentrations. Experiments in A-C are the mean value of two independent measurements (±SD). The results in D-E are from a representative experiment.

In order to demonstrate the role of a reduced PTEN level as a key indicator for the proposed combinatory treatment, we used three cell clones, that we have previously established and characterized from SKMES-1 cells [15]. In particular, we selected for the following experiments cells with overexpression of wild-type PI3K (Cl 10) or with PI3K oncogenic point mutation E545K (Cl 18) and H1047R (Cl 28). Cl 10, Cl 18 and Cl 28 cells were treated with single dose of defactinib in the absence/presence of escalating doses of buparlisib (Figure 7A–7C). Comparing the experimental combination points with the results expected by the Bliss criterion, an additive effect was observed in all the tested cell clones. Since the rationale of this combination is an increase of FAK phosphorylation in cells with altered PTEN levels, we demonstrated, that in clones with activated forms of PI3K, PTEN expression is not altered and FAK phosphorylation was unaffected both at Tyr327 and Tyr925 residues, compared to SKMES-1 control cells (Figure 7D). Moreover, combined treatment reduced both FAK and AKT phosphorylation but did not modify JNK phosphorylation (Figure 7E).

## DISCUSSION

In this study we demostrated that reduced PTEN level in SCC cells increased cell growth, migration and invasiveness, with acquisition of mesenchymal phenotype; moreover, we showed that PTEN abrogation promotes AKT and FAK activation. Lung tumors with altered PI3K signaling are not especially sensitive to monotherapy with PI3K pathway inhibitors [23]. We noted here that individual treatments with specific PI3K or FAK inhibitors (buparlisib and defactinib, respectively) were not particularly effective in cells with low PTEN levels. However, combination of buparlisib and defactinib reduced SCC cell viability, migration and invasion synergistically.

Abrogation of PTEN caused an increased growth rate as a consequence of full AKT activation, confirming that loss of PTEN expression is correlated with increased malignancy and in turn direct PI3K/AKT pathway activation. In addition, as previously reported for other malignancies [24, 25], full FAK activation was observed in cells with low PTEN levels.

We also noted that the reduction in PTEN increased migratory and invasiveness of SCC cells. This might be explained by the activation of both AKT and FAK pathways, as demonstrated by the increased expression of the active form of the GTP-binding proteins RAC1 and CDC42.

Increased malignancy/aggressiveness has been often associated with EMT, a process related to the acquisition of stem-like properties. The generation of tumor spheres has been exploited to devise a tumor model comprehensive of an increased CSC population, associated with different growth rate and response to drugs [26]. In the present study induction of EMT transition via PTEN abrogation, was confirmed by the increased expression of the mesenchymal marker Vimentin and by the reduction in the epithelial marker E-Cadherin. Moreover, the acquisition of stem-like properties, was suggested by the increased level of the stem cell marker CD133 [27], compared to cells transfected with empty vector.

To further evaluate the contribution of the reduction of PTEN to cancer progression, we evaluated the modulatory effect of PTEN expression on miR-21 levels, an oncomir involved in PTEN mRNA regulation. miR-21 has been shown to be overexpressed in different solid tumors including lung cancer [28] and its overexpression is correlated with large tumor size and advanced cancer stage. It is noteworthy that we found that PTEN abrogation increased the expression of miR-21, which in turn might be responsible for increased MMP2 mRNA expression. MiR-21 controls the expression of several MMPs, in particular MMP 2 and 9 by targeting and downregulating the inhibitor of matrix metalloproteinases TIMP3 [29, 30].

The recently published BASALT-1 study on buparlisib alone in a cohort of NSCLC patients with aberrant activation of the PI3K pathway (PI3K or PTEN point mutations and loss of PTEN expression) showed that this drug did not elicit relevant clinical effects [23]. We previously demostrated that single-agent treatments based on NVP-BEZ235 (dual PI3K/mTOR inhibitor), buparlisib or alpelisib (NVP-BYL719, a specific inhibitor of the p110α subunit of class I PI3K) were not effective in reducing cell growth of clones with altered PI3Kα expression [15]. The anti-proliferative effect of buparlisib in the P3 clone (Figure 4A and 5D), could be partially explained by considering the selectivity of buparlisib towards different members of PI3K enzyme compared to the specific p110α-inhibitor alpelisib, that did not show specific activity to cells with low PTEN level (Supplementary Figure 6). As observed in PTEN null tumors, PI3Kβ is essential for cancer growth [31]; moreover recent studies found that a subset of SCC patients is characterized by increased expression of PI3Kβ in parallel with reduction in PTEN expression [32]. The PI3K regulatory subunit P85 controls the catalytic activity of both P110α and β; in our model, abrogation of PTEN levels increased activation of this regulatory subunit of PI3K enzyme (Figure 2B). Since buparlisib inhibits both α and β isoform of PI3K, we can hypothesize that its effect could be ascribed to inhibition of the β isoform, the main responsible agent for tumor growth in PTEN null tumors.

Consistent with observations in other malignancies [25, 33], we noted increased FAK phosphorylation as a major consequence of PTEN abrogation, whereas oncogenic mutations of PI3K did not induce FAK phosphorylation. However, in contrast to the results obtained in uterine cancer, where a specific activity of GSK2256098 (FAK inhibitor) was demonstrated in PTEN-mutated compared to wild-type cancer cells [24], we found that defactinib was equally efficient in SCC cells with low PTEN levels compared to parental cells.

Interaction between buparlisib and defactinib in PTEN-deficient SCC model have yet to be elucidated. Our investigation revealed for the first time that suppression of FAK further enhanced the inhibitory effects of buparlisib in cancer cells with low PTEN levels but not in cancer cells with oncogenic mutations of PI3K. In particular, our drug interaction studies demonstrated the high performance of this combination on inhibition of cell growth in both 2D and 3D models, in addition to inhibition of cell migration and invasion. Preliminary *in vivo* data support the efficacy and safety of this combination (Supplementary Figure 5A, 5B).

These data could be explained by the reduction in AKT and FAK activation on both the phoshoporylation residues and a full reduction of JNK kinase activity. Of note, AKT is activated by PI3K alone, as demonstrated by buparlisib single-agent treatment, while FAK is not activated by PI3K, confirming that, at least in this cell line, PI3K and FAK control two upstream independent signaling axes.

As proposed by Vivanco and collaborators [34], PTEN null cells exhibited higher JNK activity independently of AKT activation: therefore, dual inhibition of AKT and JNK may be appropriate only in the context of PTEN loss, emphasizing the role of JNK in cells with low PTEN levels. Accordingly with our results, the proposed combination showed great efficacy in human T-ALL cancer [33], where PTEN mutation and inactivation are observed in 15–25% of patients. In particular, the authors underlined that FAK inactivation by PF-573228 increased the sensitivity to the PI3K inhibitor LY294002.

Therefore, the combination of buparlisib and defactinib might be exploited as a new treatment for solid tumors, such as SCC.

By exploring the clinical trials based on FAK inhibition, the combination treatment appears to be the most investigated strategy. In particular, two studies proposed a combination of defactinib with pembrolizumab (anti PD-1) with (NCT02546531) or without (NCT02758587) gemcitabine in advanced cancer; the phase 1 dose-escalation study NCT02372227 proposed the combination of defactinib with a dual PI3K/AKT inhibitor VS-5584; finally in the phase 1/2b NCT01778803 trial, defactinib is proposed in combination with paclitaxel, in ovarian cancer.

The relevance of PTEN loss in SCC prompted us to elucidate on the correlation between PTEN and FAK activation in a cohort of 51 SCC patients at different stage. The comparison of stage I-III with stage IV SCC patients in term of PTEN and p-FAK expression demonstrated a correlation with a more aggressive phenotype. Interestingly, PTEN expression has been detected in the majority of patients with early stage disease; by contrast, loss or significant reduction of PTEN expression is a common characteristic of stage IV patients. These data are in agreement with BASALT-1 [23] and IMPACT [35] studies showing that PTEN loss is a characteristic feature of advanced stage SCC. In agreement with our preclinical data, we observed an activation of FAK kinase in association with low PTEN levels, in particular in primary tumor of stage IV patients.

In stage IV patients analyzed in the IMPACT study, the pro-metastatic role of PI3K pathway activation and the retention of PTEN loss and PIK3CA mutation both in primary and brain metastasis of SCC patients has been clearely documented. Similar results were obtained in brain metastasis from melanoma [36] and breast [37] patients, where loss of PTEN function was correlated with increased AKT activity.

PTEN loss could be considered as a necessary step in the development of distant metastasis; increased activation of FAK kinase in stage IV patients could explain the pro-metastatic properties of primary tumors with loss of PTEN expression.

In conclusion, we observed a correlation between PTEN loss and FAK activation in stage IV SCC cancer patients. Preclinical data confirmed that dual treatment with specific PI3K and FAK inhibitors had a synergistic effect not only on cell growth but also on migration and invasion.

## MATERIALS AND METHODS

### Patient population

Formalin-fixed paraffin-embedded samples from patients affected by SCC were obtained from 51 patients (45 males and 6 females) aged between 47 and 85 years. The smoking status, pathological staging and clinical outcome of the disease are given in Supplementary Table 1.

### Immunohistochemical analysis

Immunohistochemistry was performed on tissue sections to detect PTEN and p-FAK expression. Samples were incubated with anti-PTEN (mouse monoclonal, clone 6H2.1, Millipore) and anti-p-FAK (rabbit monoclonal, clone 31H5L17, Thermo Fisher) antibodies, followed by DAB Detection Kit (Ventana, Roche). Finally, sections were counterstained with Mayer's hematoxylin.

PTEN and p-FAK positivity was defined by the presence of membranous (p-FAK) and cytoplasmic (PTEN) staining in neoplastic cells. A semiquantitative analysis was performed to provide a score evaluation on a tissue area of a minimum of 6.87 mm^2^ to a maximum of 457.75 mm^2^.

Samples were categorized as: (i) score 0 when no staining was detectable; (ii) score 1 when staining of tumor cells was present in less than 10% of cells; (iii) score 2 when staining involved more than 10% of cells or when weak staining was present in nearly 50% of tumor cells; (iv) score 3 when intense membrane (p-FAK) and cytoplasmic (PTEN) staining was detected in at least 50% of tumor cells.

### Cell culture

The human squamous NSCLC cell line SKMES-1 was purchased from the ATCC (Manassas, VA) and routinely maintained in MEM medium supplemented with 10% fetal bovine serum, 1 mmol/L of Na Pyruvate and 1X of NEAA.

Cl 10, Cl 18 and Cl 28 clones were previously generated from SKMES-1 cells [15] and cultured as recommended. All cell clones were cultured in a 5% CO_2_ incubator at 37°C.

### Drugs

Buparlisib (NVP-BKM120 or BKM120) alpelisib ((NVP-BYL719) were provided by Novartis Institutes for BioMedical Research (Basel, Switzerland), defactinib (VS-6063, PF-04554878) was from Selleck Biochemicals (Houston, TX). Stock solutions of 20 mM (buparlisib and defactinib) drugs were prepared in dimethylsulfoxide (DMSO), stored at −20°C and diluted in fresh medium for use. The final concentration of DMSO never exceeded 0.1% v/v.

### Stable transfection

To express artificial microRNAs (miRNAs), we used a BLOCK-iT Pol II miR RNAi system (Thermo Fisher Scientific, MA). Four double strands pre-miRNA targeting different sequences of PTEN mRNA (PTEN-miRNA1 top 5′-TGCTGTATAGGTCAAGTCTAAGTCGAGTTTTGGCCACTGACTGACTCGACTTACTTGACCTA-TA-3′, bot 5′-CCTGTATAGGTCAAGTAAGTCGAGTCAGTCAGTGG-CCAAAACTCGA CTTsAGACTTGACCTATAC-3′; PTEN-miRNA2 top 5′-TGCTGTTAGCTGGCAGACCACAAACTGTTTTG GCCACTGACTGACAGTTTGTGCTGCCAGCT AA-3′, bot 5′-CCTGTTAGCTGGCAGCACAAACTGT CAGTCAGTGGCCAAAACAGTT-TGTGGTCTG CCAGCTAAC-3′; PTEN-miRNA3 top 5′-TGCTGT GAACTTTCTTCCCGTCGT-GGTTTTGGCCACTGACTGACCACGACGGAGACAAGTTCA-3′, bot 5′-CTGTGAACTTGT-CTCCGTCGTGGTCAGTCAGTGGCCAAAACCACGACGGGAAGACAAGTTCAC-3′ and PTEN-miRNA4 top 5′-TGCTGTGTGGAAGAACTCTACTTTGAGTTTTGGCCACTGACTGAC-TCAAAGTAGTTCTTCCACA-3′, bot 5′-CCTGT GTGGAAGAACTACTTTGAGTCAGTCAGTG-GCCA AAACTCAAAGTAGAGTTCTTCCACAC-3′) or the empty vector were cloned into pcDNA ^TM^6.2-G W vector and transfected in SKMES-1 cells using and electroporation procedure, as described previously [38]. For further study, the above-mentioned transfectants were selected by fresh MEM medium containing 13 μg/mL Blasticidin (Thermo Fisher) until blasticidin-resistant colonies were identified.

### Analysis of cell viability and cell proliferation

Cell number was evaluated as previously described [39]. To evaluate the cytotoxic activity of single drugs and of the combination, the cell growth inhibitory effect of buparlisib and defactinib (0.001–10 μM) was studied using Crystal violet (CV) and Sulforhodamine B (SRB) assays in 96 wells plates (Corning Costar, New York, US). For this purpose cells were plated at 3–5 × 10^3^ cells/well and growth inhibition was expressed as the percentage versus control (vehicle-treated cells) absorbance (corrected for absorbance before drug addiction). Dose-response curves were calculated by non-linear least squares curve fitting (GraphPad PRISM, Intuitive Software for Science, San Diego, CA).

### Western blot analysis and pull-down assay

Procedures for protein extraction and protein analysis by 1-D PAGE are described elsewhere [40]. Antibodies against PTEN, p-mTOR (Ser2448), m-TOR, p-AKT (Thr308), p-AKT (Ser473), AKT, P110α-PI3K, P110β-PI3K, p-P85-PI3K (Tyr458), p-FAK (Tyr925), FAK, p-GSK3 α/β (Ser21/9), GSK3 α/β, p-SAPK/JNK (Thr183/Tyr185), SAPK/JNK, Rac1 and CDC42 were from Cell Signaling Technology (Beverly, MA). Antibody directed to p-FAK (Tyr397) was from Thermo Fisher Scientific.

The antibody against Actin was from Sigma–Aldrich (St Louis, MO). HRP-conjugated secondary antibodies were from Pierce (Rockford, IL) and chemiluminescence system (ImmobilionTM Western Chemiluminescent HRP Substrate) was from Millipore (Temecula, CA). The chemiluminescent signal was acquired by C-DiGit^®^ Blot Scanner and the spots were quantified by Image Studio^™^ Software, LI-COR Biotechnology (Lincoln, NE).

Active GTP-bound RhoA family proteins were evaluated by using Active Rho family Detection Kit from Cell Signaling Technology, accordingly to manufactures instructions [15].

### Phospho-antibody array analysis

Relative levels of activation of 46 kinase phosphorylation sites were obtained by using Proteome Profiler Human Phospho-kinase Array (Kit ARY003B from R&D System, Minneapolis, MN) according to the manufacturer's guidelines as previously reported [41]. The resulting spots were quantified using Image Studio^™^ Software, LI-COR Biotechnology (NE, US).

### Spheroid generation and growth quantification

Spheroids from transfected clones were generated using LIPIDURE^®^-COAT PLATE A-U96 (NOF Corporation, Tokjo, Japan) according to manufacturer's instruction. Briefly, 500 cells were seeded and after 1–3 days the spheroids were treated with vehicle or drugs until 14 days. The effects of the drugs were evaluated in term of volume changes using the Nikon Eclipse E400 Microscope with digital Net camera. The volume of the spheroids (V) was calculated as volume of spheres having as diameter the average of the maximum and minimum diameters [D=(Dmax+Dmin)/2; V=4/3π(D/2)^3^] obtained by measurement with Image J software [15].

### Cell migration, invasion and scratch assays

The migration and invasion assays were performed as previously described. Briefly 2 × 10^5^ cells were suspended in serum free culture medium and plated in Transwell chamber with 6.5-mm diameter polycarbonate filters (8 μm pore size, BD Biosciences, Erembodegem, Belgium) uncoated or coated with Matrigel^TM^. FBS (10%) was used as a chemoattractant in the lower chambers. After incubation for 16 h all of the non-migrated (or non-invaded) cells were removed with a cotton swab, and cells that have migrated (or invaded) through the membranes were fixed with 100% methanol, stained with hematoxylin and counted under a Phase contrast microscope [41]. The scratch assay was evaluated using the LeicaDMI300B migration station (Leica Microsystems, Eindhoven, Netherlands) integrated with the Scratch Assay 6.1 software (Digital Cell Imaging Labs, Keerbergen, Belgium); briefly cells were seeded in 96 well plate for 16 h; at the end, artificial wound tracks were created by scraping with a specific scratcher within the confluent monolayers. Cells detached were removed by washing with PBS and cultured for 16 h in complete growth medium. The migration ability of cancer cells into the wound area was assessed by comparing the pixels in the images taken at the beginning of the exposure (time 0), with those taken after 16 h. Finally the migration index (MI) was calculated according to the formula: MI=(scratch area at 0 h - scratch area at 16 h)/ scratch area at 0h.

### Quantitative real-Time PCR

Total RNA was extracted according to the Trizol-chloroform protocol. One μg RNA was retro-transcribed using the DyNAmo cDNA Synthesis Kit (Thermo Scientific, Vantaa, Finland), according to the manufacturers’ instructions. Primers to specifically amplify MMP2, Vimentin and E-cadherin were obtained from Qiagen (Venlo, Netherlands) (QT00088396, QT00095795 and QT00080143 respectively). The quantitative real-time PCR was performed in a 25-μl reaction volume containing SYBR Green Mastermix (Applied Biosystems, Forster City, CA). All reactions were performed in triplicate using the StepOne™ Real-Time PCR System instrument (Applied Biosystems). Samples were amplified using the following thermal profile: 95°C for 10 minutes, 40 cycles of denaturation at 95°C for 15 sec followed by annealing and extension at 60°C for 1 minute. Amplifications were normalized to β-Actin from Qiagen (QT00095431). The fold change was calculated by the ΔΔCT method and results were plotted as 2 ^−ΔΔCT^.

### Reverse transcription and quantitative PCR analysis of miR-21

RNA (10 ng) was reverse transcribed and the resulting cDNA was amplified using the specific Taqman MicroRNA assays (Applied Biosystems) for miR-21, RNU6B and RNU48 as endogenous controls (assay ID, 000397, 001093 and 001006, respectively). The PCRs were performed in the StepOne™ Real-Time PCR System instrument (Applied Biosystems), in accordance with the manufacturer's instructions. Data were normalized to RNU6 and RNU48 and quantification of miR-21 expression compared with untreated controls was assessed using the ΔΔCt method, plotting the results as 2 ^−ΔΔCT^ [42].

### Drug combination studies

An experimental design for comparison of buparlisib dose-response relation with that of the same drug in combination with a fixed concentration of defactinib was used and the measured responses compared to Bliss independence reference models of synergy.

A theoretical dose–response curve was calculated for combined inhibition using the equation Ebliss = EA + EB - EA × EB, where EA and EB are the percent of inhibition of cell viability versus control cells, obtained by drug A (buparlisib) and B (defactinib) alone, and Ebliss is the percent of inhibition that would be expected if the combination was exactly additive. If the experimental percent of inhibition is > Ebliss the combination is considered synergistic, if it is < Ebliss the combination is antagonistic.

Combination studies were also examined by combination index (CI) determination. CI values < 0.9 are indicative of synergistic interactions between the two agents, additive interactions are indicated by CIs between 0.9 and 1.0, whereas CIs > 1.1 indicates antagonism between the two agents. Cooperative effects were considered as interactions with a CI < 1.0. Data analysis was carried out using CalcuSyn software version 2.0 (Biosoft, Oxford, UK).

### Flow cytometry

For the determination of CD133 protein levels, cells from N1 and P3 clones were grown in 2D and 3D systems. After 10 days, 10^6^ cells were collected and incubated with Isotype control Monoclonal Mouse IgG1/R-PE or PE mouse anti-Human CD133/1 (AC133) (MiltenyiBiotecGbmH). After incubation the quantification was performed with an EPICS-XL flow cytometer, as previously described [15].

### Tumor xenografts

A mixture of 200 μL matrigel (BD Biosciences) and sterile PBS (1:1) containing 3×10^6^ P3 cells was subcutaneously injected into the flanks of BALB/c nude female mice (Charles River Laboratories, Calco, Italy). When tumor volume reached an average size of 120 mm^3^, the animals were randomly allocated into 4 groups (*n* = 6 tumors per treatment group). Buparlisib (10 mg/kg in 0.5% methylcellulose 0.1% Tween 80) and defactinib (50 mg/kg in 0.5% methylcellulose 0.1% Tween 80) were given once per day five times per week by oral gavage. Tumor xenografts were measured as previously described [15]. Comparison among groups was made using two-way repeated measures ANOVA followed by Bonferroni's posttest (to adjust for multiple comparisons). All experiments involving animals and their care were performed with the approval of the Local Ethical Committee of University of Parma, in accordance with the institutional guidelines that are in compliance with national (DL116/92) and international (86/609/CEE) laws and policies.

### Statistical analysis

Statistical analysis was carried out using GraphPad Prism 5.00 software. Statistical significance of differences among data was estimated by two-tailed Student's *t* test or by one-way ANOVA followed by Bonferroni's pos*t*-test, and *P* values are indicated where appropriate.

## SUPPLEMENTARY FIGURES AND TABLE


