# Supplementary material for: Enhanced efficacy of AKT and FAK kinase combined inhibition in squamous cell lung carcinomas with stable reduction in PTEN
Source: Oncotarget. 2017 May 23;8(32):53068–83. doi: 10.18632/oncotarget.18087 (PMC5581093; doi:10.18632/oncotarget.18087)
Supplement: Supplementary file 1 [file oncotarget-08-53068-s001.pdf]

Enhanced efficacy of AKT and FAK kinase combined inhibition in squamous cell lung carcinomas with stable reduction in PTEN

Supplementary Materials

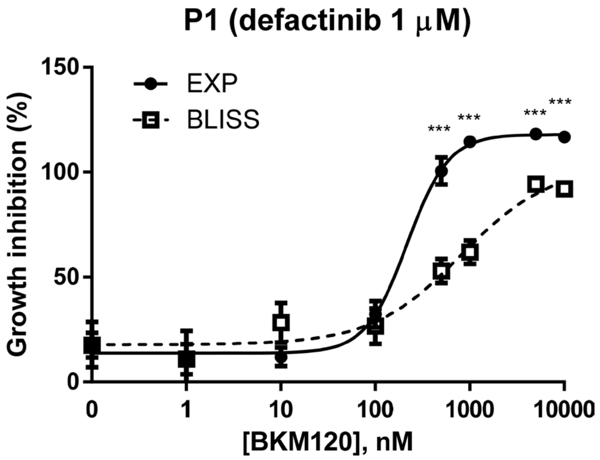

Supplementary Figure 1: The combination of buparlisib and defactinib showed synergistic effect in term of cell viability in P1 clone. Curves of the growth-inhibitory effects of the combined treatment of buparlisib with defactinib (1 μM) versus theoretical Bliss additivity curve for P1. Cells were treated with the drugs for 3 days and then cell growth was assessed using SRB assay staining as described in Material and Methods Section. Data are expressed as percent inhibition of cell proliferation versus control cells. The experiments, repeated three times, yielded similar results.***$p < 0.001$.

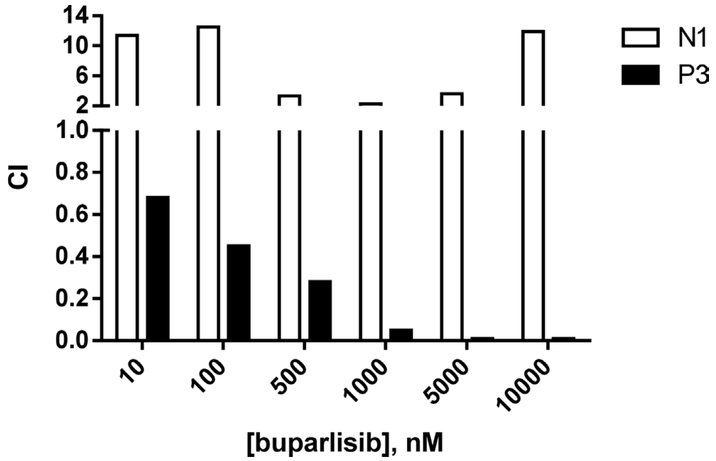

Supplementary Figure 2: Synergistic effect of buparlisib and defactinib combination in P3 cells. Representative hystogram of CI calculated as described in the Material and Method Section, after simultaneous 72-h exposure to buparlisib and defactinib 1 μM. The results are from a representative experiment.

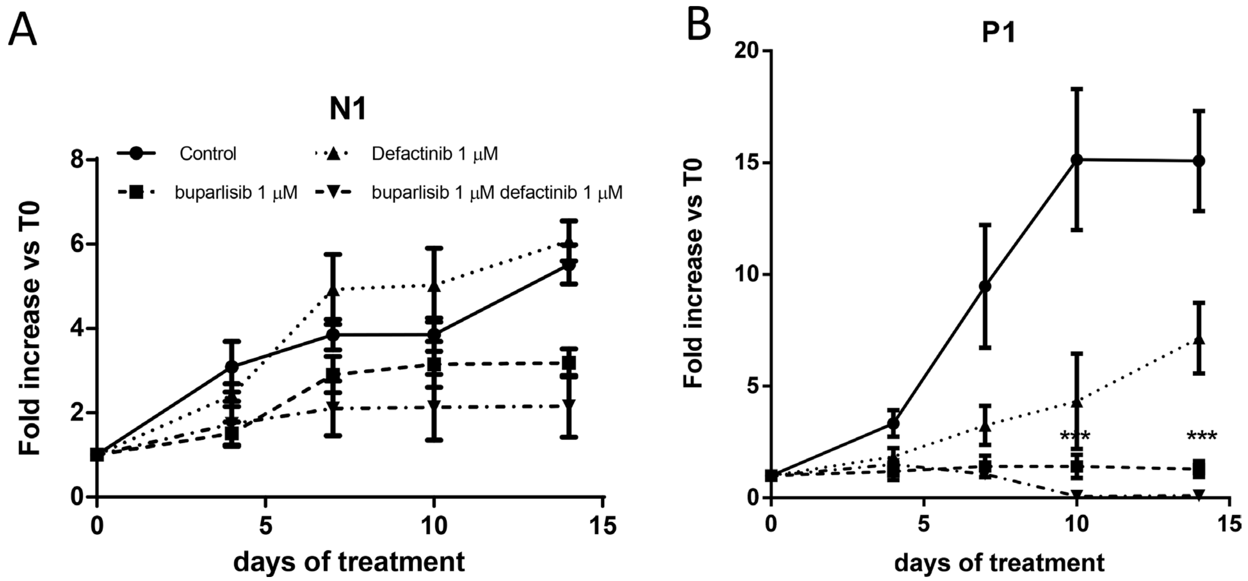

Supplementary Figure 3: The combination of buparlisib and defactinib showed synergistic effect in term of spheroid growth in P1 clone compared to control cells N1. The volume of spheroids from N1 (**A**) and P1 (**B**) clones untreated or exposed to 1 μM buparlisib or 1 μM of defactinib or their combination was measured and the Fold Increase (FI) index was calculated as the ratio between the spheroid volume at indicated time intervals and the volume at T0. ***$p < 0.001$ vs buparlisib alone.

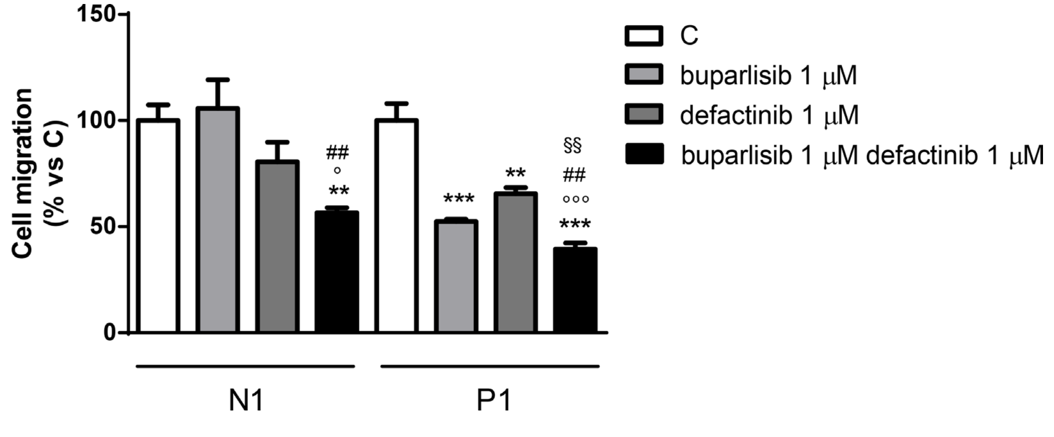

Supplementary Figure 4: The combination of buparlisib and defactinib showed synergistic effect in term of cell migration in P1 clone compared to control cells N1. Evaluation of migration in N1 and P1 clones after 16 h of treatment with buparlisib and defactinib at indicated concentrations. Columns, means of 10 fields counted; bars, SD. *$p < 0.05$, **$p < 0.01$, ***$p < 0.001$ vs Control; ##$p < 0.01$ vs buparlisib; °$p < 0.05$ vs defactinib; $^{\$\$}p < 0.01$ vs N1 combination.

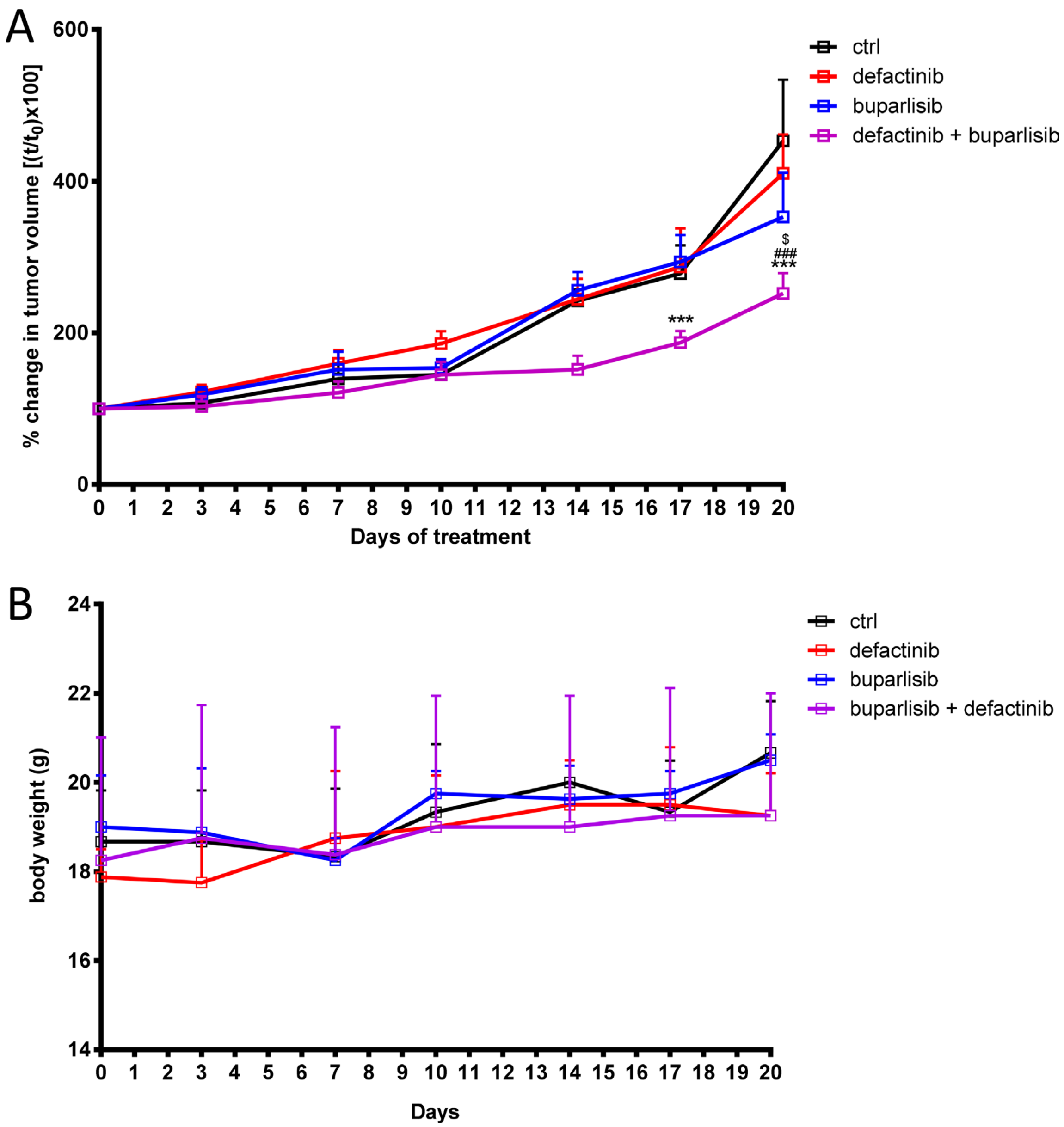

Supplementary Figure 5: Effect of buparlisib and defactinib combination in an in vivo model. (**A**) P3 cells were implanted in s.c. on BALB/C-Nude mice. Buparlisib (10 mg/Kg) and defactinib (50 mg/Kg) were given once per day five times per week by oral gavage. Data are expressed as the percentage of change in tumor volume ± SEM of 6 tumors per group. (**B**) Effect of the treatment of mice with vehicle (CTRL), buparlisib, defactinib, or combination administered orally five days per week on animal body weight. Data are expressed as body weight ± SEM. $^{***}p < 0.001$ vs ctrl; $^{###}p < 0.001$ vs defactinib; $^{\$}p = 0.0716$ vs buparlisib. Two-way ANOVA followed by Bonferroni post-test.

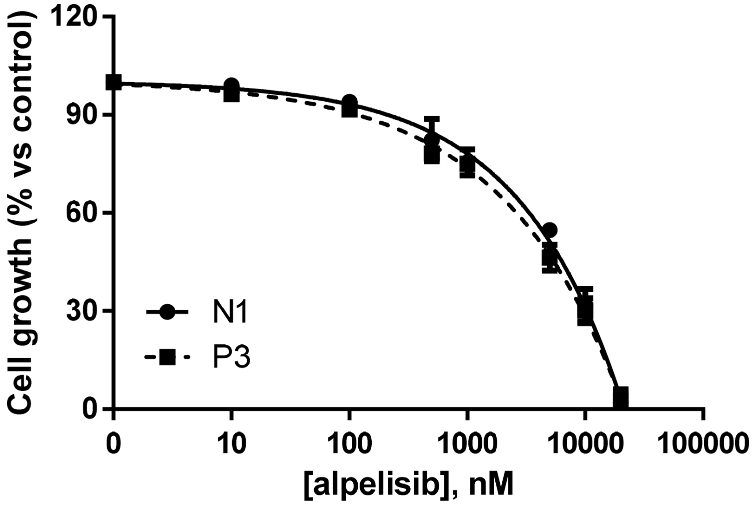

Supplementary Figure 6: Alpelisib has similar effects in inhibiting cell growth in N1 and P3 cells. N1 and P3 cells. were treated with increasing concentrations of the specific inhibitor of PI3K-α isoform alpelisib and cell growth was determined by SRB assay. Experiment is the mean value (±SD) of three independent measurements.

Supplementary Table 1: Patient characteristics and relative PTEN and phospho-FAK expressions

ID	Age, years	Sex	Smoker	Stage	Clinical Outcome	PTEN	p-FAK
1	70	M	Yes	IIIa	Resected and disease free	1	2
2	64	M	Yes	IIIa	Resected and disease free	0	2
3	67	M	Yes	IIa	Resected and disease free	2	3
4	69	M	Yes	Ia	Resected and disease free	0	0
5	77	M	Ex	Ib	Resected and disease free	0	2
6	76	M	Ex	IIa	Resected and disease free	3	2
7	72	M	Ex	IIa	Resected and disease free	3	1
8	77	F	Yes	IIa	Resected and disease free	0	0
9	81	M	Ex	IIb	Resected and disease free	1	2
10	73	M	Ex	IIb	Resected and disease free	0	1
11	70	M	Ex	Ib	Resected and disease free	1	3
12	82	M	Ex	Ib	Resected and disease free	3	1
13	66	F	Yes	IIa	Resected and disease free	0	0
14	72	M	Yes	IIa	Resected and disease free	0	2
15	81	M	Ex	IIIa	Resected and disease free	1	0
16	56	M	Ex	IIIa	Resected and relapsed	0	1
17	75	M	Ex	Ib	Resected and relapsed	3	0
18	54	M	Yes	IIIa	Resected and relapsed	0	1
19	76	M	Ex	Ia	Resected and relapsed	3	2
20	66	M	Yes	Ia	Resected and relapsed	0	2
21	68	M	Ex	IIIa	Resected and relapsed	2	1
22	73	M	Ex	IIa	Resected and relapsed	0	2
23	69	M	Yes	IIb	Resected and relapsed	1	2
24	74	F	Yes	Ia	Resected and relapsed	1	1
25	70	M	Yes	IIa	Resected and relapsed	1	2
26	71	M	Yes	IV	Metastatic	1	2
27	55	M	Yes	IV	Metastatic	0	2
28	73	M	Yes	IV	Metastatic	1	3
29	70	M	Yes	IV	Metastatic	0	3
30	84	M	Yes	IV	Metastatic	0	3
31	65	M	Yes	IV	Metastatic	1	1
32	61	F	Yes	IV	Metastatic	0	3
33	77	M	Yes	IV	Metastatic	0	2
34	83	M	Yes	IV	Metastatic	0	3
35	65	M	Ex	IV	Metastatic	1	2
36	78	M	Yes	IV	Metastatic	1	1
37	70	F	Yes	IV	Metastatic	1	1
38	74	M	Yes	IV	Metastatic	0	3
39	79	M	Yes	IV	Metastatic	0	3
40	77	M	Yes	IV	Metastatic	0	3
41	81	M	NA	IV	Metastatic	0	3
42	71	M	NA	IV	Metastatic	1	2
43	85	M	NA	IV	Metastatic	1	2
44	47	M	NA	IV	Metastatic	3	3
45	72	M	Ex	IV	Metastatic	0	0
46	69	M	Yes	IV	Metastatic	3	1
47	75	M	NA	IV	Metastatic	1	2
48	64	M	NA	IV	Metastatic	3	1
49	77	M	NA	IV	Metastatic	1	0
50	71	M	NA	IV	Metastatic	2	3
51	69	F	NA	IV	Metastatic	0	2
